# Mapping the Contrast Sensitivity of the Visual Field With Bayesian Adaptive qVFM

**DOI:** 10.3389/fnins.2020.00665

**Published:** 2020-07-07

**Authors:** Pengjing Xu, Luis A. Lesmes, Deyue Yu, Zhong-Lin Lu

**Affiliations:** ^1^College of Optometry, The Ohio State University, Columbus, OH, United States; ^2^Adaptive Sensory Technology, Inc., San Diego, CA, United States; ^3^Division of Arts and Sciences, NYU Shanghai, Shanghai, China; ^4^Center for Neural Science and Department of Psychology, New York University, New York, NY, United States; ^5^NYU-ECNU Institute of Brain and Cognitive Science at NYU Shanghai, Shanghai, China

**Keywords:** Bayesian adaptive testing, automated perimetry, visual-filed map, peripheral vision, contrast sensitivity, active learning, Sloan letters

## Abstract

Current clinical evaluation, which focuses on central vision, could be improved through characterization of residual vision with peripheral testing of visual acuity, contrast sensitivity, color vision, crowding, and reading speed. Assessing visual functions in addition to light sensitivity, a comprehensive visual field map (VFM) would be valuable for detecting and managing eye diseases. In a previous study, we developed a Bayesian adaptive qVFM method that combines a global module for preliminary assessment of the VFM's shape and a local module for assessment at individual retinal locations. The method was validated in measuring the light sensitivity VFM. In this study, we extended the qVFM method to measure contrast sensitivity across the visual field. In both simulations and psychophysics, we sampled 64 visual field locations (48 x 48 deg) and compared the qVFM method with a procedure that tested each retinal location independently (qFC; Lesmes et al., 2015). In each trial, subjects were required to identify a single optotype (size: 2.5 x 2.5 deg), one of 10 filtered Sloan letters. To compare the accuracy and precision of the two methods, three simulated eyes were tested in 1,280 trials with each method. In addition, data were collected from 10 eyes (5 OS, 5 OD) of five normal observers. For simulations, the average RMSE of the estimated contrast sensitivity with the qVFM and qFC methods were 0.057 and 0.100 after 320 trials, and 0.037 and 0.041 after 1,280 trials [all in log10 units, represent as *log(sensitivity)*], respectively. The average SD of the qVFM and qFC estimates were 0.054 and 0.096 after 320 trials, and 0.032 and 0.041 after 1,280 trials, respectively. The within-run variability (68.2% HWCIs) were comparable to the cross-run variability (SD). In the psychophysics experiment, the average HWCI of the estimated contrast sensitivity from the qVFM and qFC methods across the visual field decreased from 0.33 on the first trial to 0.072 and 0.16 after 160, and to 0.060 and 0.10 after 320 trials. The RMSE between the qVFM and qFC estimates started at 0.26, decreased to 0.12 after 160 and to 0.11 after 320 qVFM trials. The qVFM provides an accurate, precise, and efficient mapping of contrast sensitivity across the entire visual field. The method might find potential clinical applications in monitoring vision loss, evaluating therapeutic interventions, and developing effective rehabilitation for visual diseases.

## Introduction

A comprehensive characterization of peripheral vision with assessment of the Visual Filed Map (VFM) is crucial for monitoring the status of vision loss, for developing and providing effective rehabilitation interventions (Sunness et al., 1995; Fletcher and Schuchard, 1997; Markowitz and Muller, 2004), and for obtaining projections of potential benefits from interventions (Massof and Rubin, 2001; Strasburger et al., 2011).

As a part of the clinical ophthalmic diagnostic procedure, the VFM of light sensitivity is assessed by the majority of eye care practitioners, mostly using the standard automated perimetry (SAP) (Dreyer, 1993; Johnson et al., 2011). Assessment of the VFM of many other visual functions, such as contrast sensitivity (Daitch and Green, 1969; Swanson et al., 2014), visual acuity (VA, 1965; Thompson et al., 1982), color vision (Carlow et al., 1976; Hart et al., 1984; Sample and Weinreb, 1990, 1992), reading speed (Ramulu et al., 2009; Yu et al., 2010), and crowding (Balas et al., 2009; Levi and Carney, 2009), is difficult and rarely used in the clinic. In fact, results from the standard automated perimetry (SAP) are noisy (Stewart and Hunt, 1993; Keltner et al., 2000). Precise and accurate VFM assessments of visual functions are time consuming with existing methods (Artes et al., 2002; Weinreb and Kaufman, 2009, 2011). A number of new perimetric methods have been developed and could potentially provide helpful clinical information, but have not sufficiently validated for routine clinical use (Johnson et al., 2011; Strasburger et al., 2011; Keltgen and Swanson, 2012; Swanson et al., 2014).

In a previous study, we developed a novel Bayesian adaptive testing method, the qVFM method, that combines a global module for preliminary assessment of the VFM's shape and a local module for assessing individual visual field locations to provide an efficient and precise assessment of the VFM (Xu et al., 2019). In its first implementation, we applied the qVFM method to assess the light sensitivity visual field map with a Yes/No paradigm. Computer simulations and a psychophysical validation study both showed that the qVFM method could provide an accurate, precise and efficient assessment of light sensitivity VFM.

In this study, we implemented the qVFM method in a 10-alternative forced-choice (10AFC) letter identification paradigm to measure contrast sensitivity (CS) across the visual field to provide an assessment of the contrast sensitivity visual field map.

As a clinical measure, contrast sensitivity predicts functional vision better than many other visual diagnostics (Comerford, 1983; Jindra and Zemon, 1989; Ginsburg, 2003; Faye, 2005). Deficits in contrast sensitivity accompany many visual diseases, including amblyopia (Hess and Howell, 1977; Bradley and Freeman, 1981; Kiorpes et al., 1999; Xu et al., 2006; Qiu et al., 2007), glaucoma (Ross et al., 1984; Stamper, 1984; Hot et al., 2008), optic neuritis (Zimmern et al., 1979; Trobe et al., 1996), diabetic retinopathy (Della Sala et al., 1985; Sokol et al., 1985), Parkinson's disease (Bulens et al., 1986; Bodis-Wollner et al., 1987; Mestre et al., 1990), and multiple sclerosis (Regan et al., 1981, 1982; Hess and Plant, 1985; Travis and Thompson, 1989; Regan and Hamstra, 1991). Such deficits are evident even when acuity and/or light sensitivity perimetry tests appear normal (Jindra and Zemon, 1989; Woods and Wood, 1995). Contrast sensitivity is also an important outcome measure of refractive and cataract surgery (Ginsburg, 1987, 2006; Applegate et al., 1998, 2000; McLeod, 2001; Bellucci et al., 2005), and potential rehabilitation programs for macular degeneration (Loshin and White, 1984), myopia (Tan and Fong, 2008), and amblyopia (Polat et al., 2004; Li et al., 2005, 2009; Zhou et al., 2006; Huang et al., 2008). On the other hand, although the literature has documented the importance of contrast sensitivity test, the current in-clinic contrast sensitivity exams mostly consist of contrast sensitivity measurements in fovea, e.g., the Pelli-Robson chart (Pelli and Robson, 1988), which can only provide a limited contrast sensitivity assessment of residual spatial vision for ophthalmic patients (Elliott and Whitaker, 1992).

Our new implementation of the qVFM method was based on the qFC procedure, originally developed to measure contrast sensitivity with forced-choice paradigms at a single visual location (Hou et al., 2015; Lesmes et al., 2015). Here, we integrated the qFC procedure with the qVFM method to assess contrast sensitivity across the visual field. In the rest of this paper, we first briefly describe the 10AFC implementation of the qVFM method, then computer simulations, and finally a psychophysical validation experiment.

## qVFM With 10-AFC

The qVFM method consists of three major modules (Xu et al., 2019; see Appendix C for more details):

1. The global module, which assesses the shape of the VFM through a Bayesian adaptive procedure to estimate the posterior distributions of the parameters of a tilted elliptic paraboloid function (TEPF):
  (1) $$\begin{array}{l} \tau \left(x,y\right)=EPZ-{\left(\frac{x}{EPA}\right)}^{2}-{\left(\frac{y}{EPB}\right)}^{2}+SLA*x+SLB*y \end{array}$$
  where *EPZ* is the contrast sensitivity at the fovea, *EPA* is the bandwidth (latus rectum) in the horizontal direction, *EPB* is the bandwidth in the vertical direction, *SLA* is the tilt level in the horizontal direction, and *SLB* is the tilt level in the vertical direction. The height of the TEPF, τ(x, y), defines the contrast sensitivity (1/contrast) at a fixed *d*′ = 1.5 level at visual field location (x, y).
2. The switch module, which evaluates the rate of information gain in the global module and determines when to switch to the local module, and, at the point of the switch, generates the prior distribution of the visual function (e.g., light sensitivity, contrast sensitivity) at each visual field location based on the posterior from the global module.
3. The local module, which provides independent assessment of visual function at each visual field location using another Bayesian adaptive procedure that determines the location and stimulus parameters of test stimulus in each trial based on the relative information gain across locations.

In the global module, a probability density function, $p\left(\vec{\theta }\right)$, where $\vec{\theta }=\left(EPZ,\;EPA,\;EPB,\;SLA,\;SLB\right),\;$is defined over the parameter space of the TEPF. The initial prior distribution *p*_*t* = 0_($\vec{\theta }$) represents foreknowledge of model parameters before any data collection (trial *t* = 0). A stimulus space, which includes all possible stimulus intensities and stimulus locations (x, y), is also defined in the qVFM procedure. The local module starts with a prior distribution in each retinal location. In both the global and local modules, a one-step-ahead search strategy is used to determine the optimal stimulus in the next trial that would lead to the maximum information gain (equivalent to the minimum expected entropy), and the selection of optimal stimulus location and intensity is always based on the total expected entropy across all the visual field locations. Using Bayesian update and optimal stimulus selection (Kontsevich and Tyler, 1999; Lesmes et al., 2006, 2010, 2015; Lu and Dosher, 2013), the qVFM updates the posterior distribution of the parameters based on subject's response in each trial to estimate the shape of the VFM in the global module or the individual parameters of each location in the local module.

In a previous paper (Xu et al., 2019), we implemented the qVFM method with a Yes/No task. In the new 10AFC implementation, we kept the general algorithm unchanged except the likelihood function, which was based on the *d*′ psychometric function for Yes/No in the earlier implementation of the method. In a 10-AFC task, the *d*′ psychometric function (i.e., perceptual sensitivity for a given stimulus contrast *s)* at each visual field location (x, y), can be modeled as (Foley and Legge, 1981; Legge et al., 1987; Hou et al., 2015):

(2) $$\begin{array}{l} d^{\prime }\left(s,x,y\right)=1.5{\left(\frac{\tau \left(x,y\right)}{s}\right)}^{\gamma } \end{array}$$

where *s* is the reciprocal of the contrast of the stimulus (i.e., 1/contrast), τ(*x, y*) is the contrast sensitivity at location (x,y), γ is the steepness of the *d*′ psychometric function. Plotted on log-log axes, this function is linear over the contrast of the stimulus. Following previous studies (Foley and Legge, 1981; Lu and Dosher, 1999; Hou et al., 2015; Lu et al., 2019), we set γ = 2.35 in the current implementation of the qVFM. Based on signal detection theory (Gu and Green, 1994; Klein, 2001), the probability of correctly identifying the target in an m-alternative forced choice (m-AFC) identification task is a function of the corresponding *d*′ (Hacker and Ratcliff, 1979):

(3) $$\begin{array}{l} P\left(s,x,y\right)=\int_{-\infty }^{+\infty }\phi \left(t-d^{\prime }\left(s,x,y\right)\right)\Phi^{m-1}\left(t\right)\;dt \end{array}$$

where ϕ() is the probability density function of the standard normal distribution, Φ() is the cumulative probability density function of the standard normal distribution, m is the number of alternatives in the m-AFC task (which is 10 in this study), and *d*′*(s, x, y)* is the *d*′ value for a stimulus *s* at visual field location *(x,y)*. In an m-AFC task, the observer compares the internal responses of the target with those of the m-1 non-target. The probability density of obtaining an internal response *t* from the target stimulus is ϕ(*t*−*d*′(*s, x, y*)); the probability density of obtaining an internal response *t* that is greater than all the m-1 non-target responses is Φ^m−1^*(t);* and, according to the max decision rule, the probability of correctly identifying the target, *P*(*s, x, y*), is the probability that all possible internal responses of the target are greater than those from the m-1 non-targets, which is the product of the two probability density functions integrated over all the possible values of *t* (Lu and Dosher, 2013).

In addition, we assume a fixed lapse rate ε for human observers (Klein, 2001; Wichmann and Hill, 2001; Lesmes et al., 2015):

(4) $$\begin{array}{l} P^{\prime }\left(s,x,y\right)=\frac{1}{10}\varepsilon +\left(1-\varepsilon \right)P\left(s,x,y\right) \end{array}$$

where *P(s,x,y)* is the psychometric function without lapse (Equation 3). In the qVFM method, ε is set to 0.03 (Wichmann and Hill, 2001; Lesmes et al., 2010). Equation (4) defines the likelihood function that completely describes the probability of 10AFC target identification across all visual field locations and contrast levels in the qVFM method.

## Simulations

### Methods

To evaluate the performance of the qVFM procedure for observers with a range of performance, we simulated three observers asked to perform a 10AFC letter identification task in 64 retinal locations. The parameters of the three simulated observers were chosen to approximate those of the observers in our psychophysical validation study, shown in Table 1. The blind spot of all simulated observers was at (−15 degree, −3 degree).

**Table 1:** Parameters of the three simulated observers.

**Simulation**	**EPA**	**EPB**	**EPZ**	**SLA**	**SLB**
Observer 1	72	54	0.60	0.003	0.005
Observer 2	54	48	1.2	0.001	0.003
Observer 3	61	55	0.85	0.002	0.004

*The unit of EPA and EPB is degree/$\sqrt{log(sensitivity)}$, unit of EPZ is log(sensitivity), and unit of SLA and SLB is log(sensitivity)/degree*.

In the qVFM method, the parameter space includes 30 linearly spaced EPA values [from 36.0 to 96.0 degree/$\sqrt{log\left(sensitivity\right)}$], 30 linearly spaced EPB values [from 36.0 to 96.0 degree/$\sqrt{log\left(sensitivity\right)}$], 50 linearly spaced EPZ values [from 0.25 to 1.4 *log*(*sensitivity*)], 20 linearly spaced SLA values [from −0.015 to 0.015 *log*(*sensitivity*)/degree] and 20 linearly spaced SLB values [from −0.016 to 0.016 *log*(*sensitivity*)/degree]. The broad parameter space ensures robust assessment of a wide range of patient populations and avoids effects of extreme values—the tendency to bias toward the center of the parameter space when the observer's true parameter values are close to the boundary of the space.

For each of the five qVFM parameters, the priors were defined by a hyperbolic secant (sech) function (King-Smith and Rose, 1997). For each qVFM parameter, θ_*i*_, for i = 1, 2, 3, 4, 5, the mode of the marginal prior *p(*θ_*i*_*)* was defined by the best guess for that parameter based on a pilot study, θ_*i,guess*_, and the width was defined by the confidence, θ_*i,confidence*_:

(5) $$\begin{array}{l} P\left(\theta_{i}\right)=sech\left(\theta_{i,confidence}\times \left(\theta_{i}-\theta_{i,\;guess}\right)\right) \end{array}$$

The priors were log-symmetric around θ_*i, guess*_, whose values for the respective parameters were: EPA = 60 (degree/$\sqrt{log\left(sensitivity\right)}$), EPB = 54 (degree/$\sqrt{log\left(sensitivity\right)}$), EPZ = 0.90 (*log*(*sensitivity*)), SLA = 0.002 (*log*(*sensitivity*)/degree), and SLB = 0.003 (*log*(*sensitivity*)/degree). For θ_*confidence*_ of each parameter, the value was set to 3.1 for EPA, 2.6 for EPB, 3.4 for EPZ, 5.2 for SLA, 4.5 for SLB. The joint prior was defined as the normalized product of the marginal priors, which resulted in a relatively moderate informative prior for the three simulated observers in our study.

The stimulus space includes an 8 x 8 grid of retina locations (48 x 48 degree) and log-linearly spaced contrast values [between 0.05 to 1.0, corresponding to 0 to 1.3 *log*(*sensitivity*)]: with 60 values in the global module and 120 contrast values in the local module.

We compared the performance of the full qVFM procedure that has all three modules with a reduced qVFM procedure that has only the local module in 1,000 repeated simulations of 1,280 trials each. The priors in the reduced qVFM was generated from the prior of the global module of the full qVFM. In other words, the two methods are equated before the first trial.

### Metrics of Evaluation

Accuracy is a measure of how much the estimate deviate from the truth on average, and precision is a measure of the variability of repeated estimates. We quantify accuracy using the root mean squared error (RMSE) of the estimated contrast sensitivities across all 64 visual field locations. The RMSE after the i-th trial can be calculated as:

(6) $$\begin{array}{l} RMSE_{i}^{simulation}=\sqrt{\frac{\sum_{k}\sum_{j}{\left(\tau_{ijk}-\tau_{k}^{true}\right)}^{2}}{J\times K}} \end{array}$$

where τ_*ijk*_ is the estimated sensitivity at the *k*-th VF location after *i* trials in the *j*-th run, and $\tau_{k}^{true}$ is the true sensitivity at that location.

Precision is defined as the inverse of the variability of the estimates. Two methods were used to assess the precision of the qVFM method. The first is based on the standard deviation of repeated measures:

(7) $$\begin{array}{l} SD_{i}=\sqrt{\frac{\sum_{k}\sum_{j}{\left(\tau_{ijk}-mean\left(\tau_{ijk}\right)\right)}^{2}}{J\times K}} \end{array}$$

Another measure of precision is the average half width of the credible interval (HWCI) of the posterior distribution of the estimated sensitivities across retina locations. The 68.2% credible interval represents the range within which the actual value lies with 68.2% probability. Since researchers typically do not repeat an experiment many times for the same observer, the HWCI of the posterior distribution is a very important index of precision that can be obtained with a single run of the qVFM procedure (Hou et al., 2015).

### Results

A simulation of the qVFM and qFC methods based on the parameters of the simulated observer 1 is shown in Supplementary Movie 1. The simulation program can be downloaded from GitHub (https://github.com/hvxpj/qVFM_Demo/issues/1#issue-604728692). The GUI allows users to adjust the parameters of the simulated observers and prior used in the qVFM method.

**Figure 1:**
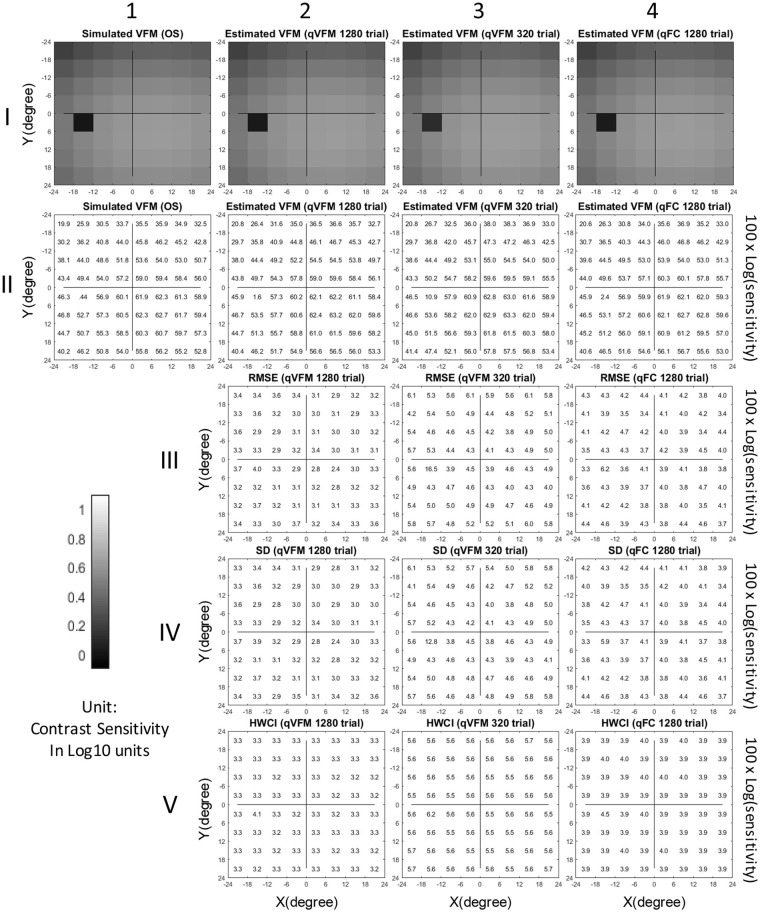
Simulation results I (Observer 1): The contrast sensitivity VFM of the simulated observer (I,1; II,1); The estimated VFM obtained with the qVFM method after 1,280 trials (I,2; II,2) and 320 trials (I,3; II,3); The estimated VFM obtained with the qFC method (I,4; II,4); And the corresponding RMSE^simulation^ (III), standard deviation (IV), and HWCI (V) of the estimates.

The estimated VFMs of the three simulated observers, obtained with the qVFM and qFC methods, are shown in Figure 1 (simulated observer 1) and Figures A1, A2 (simulated observers 2 and 3) in Appendix A.

In characterizing spatial vision, the area under the log contrast sensitivity function is often used as a summary metric (Applegate et al., 1998, 2000; Oshika et al., 1999, 2006; van Gaalen et al., 2009; Hou et al., 2010; Lesmes et al., 2010; Jia et al., 2014; Dorr et al., 2015; Zheng et al., 2018). Here, we used the volume under the surface of the VFM (VUSVFM) to provide a summary metric of the entire visual field.

**Figure 2:**
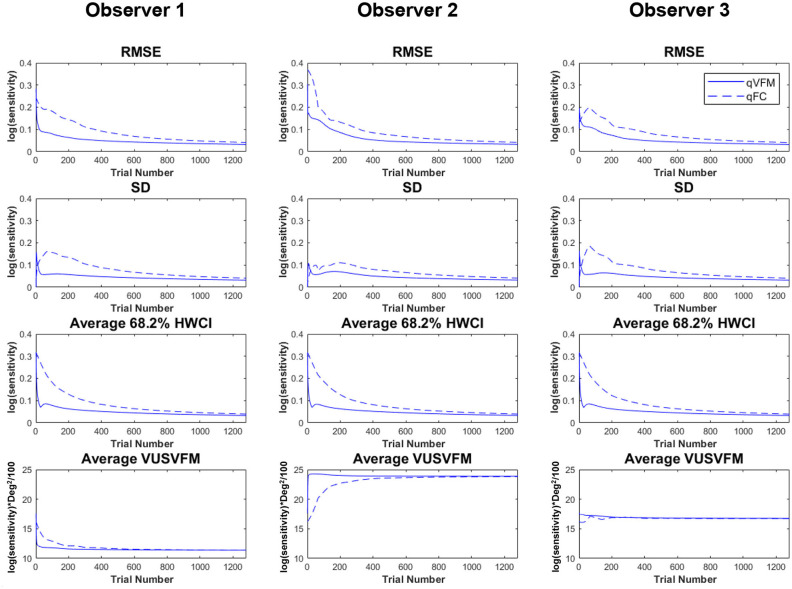
Simulation results II. The first three rows show the RMSE^simulation^, standard deviation, and average 68.2% HWCI of the estimated contrast sensitivities across 64 locations and 1,000 runs. The fourth row shows the average volume under the surface of the VFM (VUSVFM) across 1,000 runs. The true VUSVFM is 11.3 for observer 1, 23.8 for observer 2 and 16.7 for observer 3 (unit: *log(sensitivity)*) x degree^2^/100.

Figure 2 shows the RMSE^simulation^, standard deviation, average 68.2% HWCI and VUSVFM of the estimated contrast sensitivities obtained from the qVFM and qFC methods for the three simulated observers over 1,280 trials. In log10 units [represent as *log*(*sensitivity*)], the average RMSE^simulation^ of the three simulated observers started at 0.24 for both the qVFM and qFC methods. It decreased to 0.057 and 0.10 in the qVFM and qFC methods after the first 320 trials, and to 0.037 and 0.041 in the two methods after 1,280 trials, respectively. The SD of the estimated sensitivities was 0.054 in the qVFM method and 0.096 in the qFC method after 320 trials, which decreased to 0.032 in the qVFM method and 0.041 in the qFC method after 1,280 trials. The average 68.2% HWCI of the estimated sensitivities also decreased with trial number. It started at 0.32 in both the qVFM and qFC methods, decreased to 0.055 in the qVFM method and 0.094 in the qFC method after the first 320 trials, and to 0.033 in the qVFM method and 0.039 in the qFC method after 1,280 trials.

For the qVFM method, the switch from the global module to the local module occurred between 31 and 69 trials, with the mean around 41 trials and standard deviation of 9.3 trials. From Figure 2, we can see that the global module acted very efficiently in reducing random errors and uncertainties in the beginning of the measurement.

The simulations showed that the estimated VFM from both the qVFM and qFC method could reach high accuracy and precision in 1,280 trials. The qVFM method could however converge much quicker and achieve good accuracy and precision in a much shorter period of time comparing to the qFC method. To achieve 0.1 log(*sensitivity*) accuracy and 0.1 *log*(*sensitivity*) precision, on average, the qVFM method only took 106 trials, whereas the qFC method needed 334 trials.

## Psychophysical Validation

### Methods

#### Participants

We collected data from ten eyes (5 OS, 5 OD) of 5 normal (3 male and 2 female) subjects, including four naïve observers (Subject 2–Subject 5) and one of the authors (Subject 1). All subjects were between 32 and 39 years of age.

#### Apparatus

The psychophysical experiment was conducted on an IBM PC compatible computer, running Matlab programs with *PsychToolbox* extensions (Brainard and Vision, 1997; Pelli, 1997). Subjects viewed the stimuli monocularly with natural pupil at a viewing distance of 30 cm in a dimly lighted room. The stimuli were displayed on a Samsung 55-inch monitor [Model: UN55FH6030, Clear Motion Rate (CMR) of 240], with a screen size of 120.6 x 67.8 cm, corresponding to a field of view 127.0 x 97.0 degrees for the subjects, a screen resolution of 1920 x 1080 pixels, a refresh rate of 60 Hz, and a background luminance at 47 cd/m^2^. A chin–forehead rest was used to minimize head movements during the experiment.

#### Stimuli

**Figure 3:**
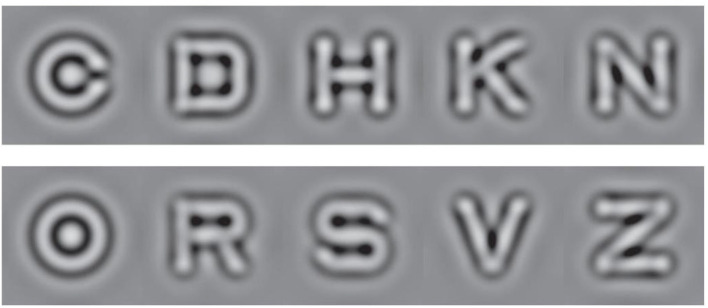
The 10 filtered Sloan letters used in the study.

Ten Sloan letters, filtered with a raised cosine filter and octave bandwidth (central spatial frequency: 1.2 cycles per degree), served as stimuli (Figure 3). The contrast of the letters varied between 0.05 to 1, corresponding to 0 to 1.3 *log*(*sensitivity*).

#### Design and Procedure

**Figure 4:**
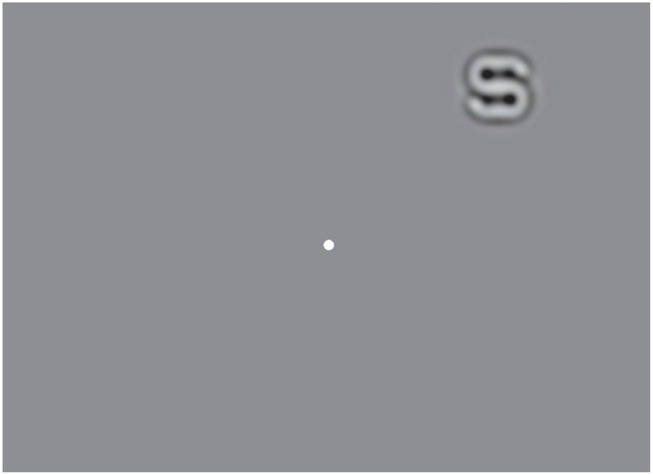
Illustration of the stimulus layout in the psychophysical experiment. A filtered Sloan letter was displayed for 200 ms at one of the 8×8 possible retinal locations on the screen. Subjects were asked to fixate on the center dot and report which letter was present.

In each trial, a single optotype (size: 2.5 x 2.5 degree) was presented for 200 ms in one of the 8 x 8 possible retina locations, evenly distributed in a 48 x 48-degree visual field (Figure 4). Subjects were asked to identify the letter. On each trial, the contrast and location of the stimulus was adaptively selected. The inter-trial interval was set to 1.2 s.

Each eye was tested in four sessions, each consisting of an independent 320-trial qVFM assessment and 320 qFC trials, with the two types of trials randomly mixed.

### Results

**Figure 5:**
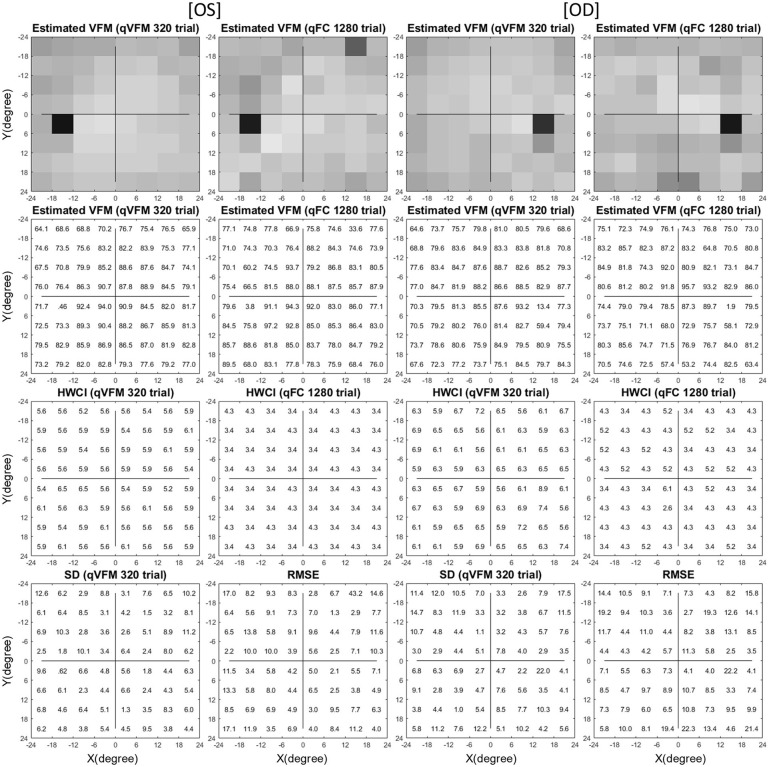
Experimental result I (Subject 1, OS and OD). The estimated contrast sensitivity VFMs are presented in the first row with colormaps and second row with numerical values (unit: 100 x *log(sensitivity)*). For each visual field location of the estimated VFM, the 68.2% HWCI is presented in the third row, the standard deviation of the estimated contrast sensitivity from the 4 repeated qVFM assessments and the RMSE^eyes^ between the qVFM and qFC estimates are presented in the fourth row. The results obtained from OS are displayed in the first and second columns, and OD in the third and fourth columns, respectively. The results from the qVFM and qFC methods are displayed in different columns.

The estimated VFMs of the 10 tested eyes from both the qVFM and qFC methods are shown in Figure 5 (Subject 1) and Figures B1–B4 (Subject 2-5, in Appendix B).

**Figure 6:**
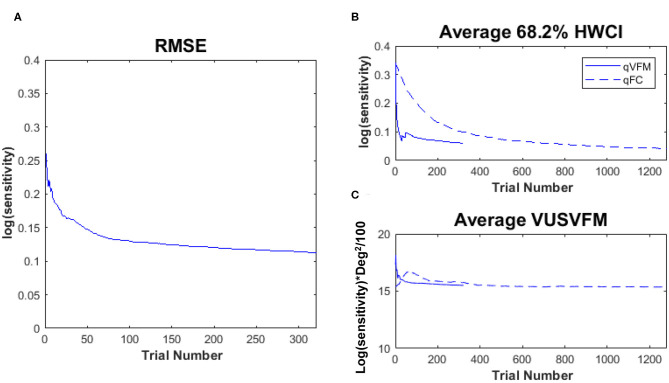
Experimental results II. **(A)** RMSE^eyes^ of the estimated sensitivities from qVFM as a function of trial number, using estimated sensitivities from 1,280 qFC trials as the “truth.” **(B)** Average 68.2% HWCI of the estimated sensitivities across 64 locations and 10 eyes. **(C)** Average VUSVFM across 10 eyes. Results from the qVFM method are shown in solid lines, and results from the qFC method are shown in dashed lines.

The agreement between the estimated VFMs from the qVFM and qFC was evaluated by the root mean squared error (RMSE) of the estimated contrast sensitivities across all 64 retina locations:

(8) $$\begin{array}{l} RMSE_{i}^{eyes}=\sqrt{\frac{\sum_{l}\sum_{k}\sum_{j}{\left(\tau_{ijkl}^{qVFM}-{\;\tau }_{kl}^{qFC}\right)}^{2}}{J\times K\times L}} \end{array}$$

where $\tau_{ijkl}^{qVFM}$ is the estimated contrast sensitivity from the qVFM method in the *k*-th VF location of the *l*-th eye after *i* trials in the *j*-th session, and ${\;\tau }_{kl}^{qFC}$ is the estimated contrast sensitivity from the qFC method in the *k*-th VF location of the *l*-th eye after 1,280 trials. The average RMSE^eyes^ (in *log*(*sensitivity*) units) started at 0.26 on the first qVFM trial and decreased to 0.12 after 160 qVFM trials and to 0.11 after 320 qVFM trials across all test sessions and eyes (Figure 6A). That the decreasing RMSE^eyes^ estimates is a function of trial number suggests that the accuracy of qVFM increased with number of test trials.

The average 68.2% HWCI of the estimated contrast sensitivities [in log10 units, represent as *log*(*sensitivity*)] across all 10 eyes and 64 retina locations decreased from 0.33 before the first qVFM trial to 0.072 after 160 qVFM trials and 0.060 after 320 qVFM trials. The average 68.2% HWCI of the estimated contrast sensitivities decreased from 0.33 before the first qFC trial to 0.16 after 160 qFC trials, 0.10 after 320 qFC trials, and 0.041 after 1,280 qFC trials (Figure 6B). The results suggest that the precision of the estimated sensitivities from the qVFM and qFC methods increased with trial number, and reached 0.1 *log*(*sensitivity*) in about 17 and 325 trials, respectively.

For the qVFM method, the switch from the global module to the local module occurred between 31 and 70 trials, with the mean around 41 trials and a standard deviation of 9.8 trials across all 10 eyes, consistent with the simulations. The rapid convergence of the VFM estimates by the global module (the average 68.2% HWCI) is evident in Figure 6B.

Figure 6C presents the average estimated VUSVFM of 10 eyes as a function of trial number for qVFM and qFC. The estimated VUSVFM from the two methods was less than 0.6% different after 320 trials. The agreement of these estimates implies that the VUSVFM can be a useful metric of the overall visual filed map.

**Figure 7:**
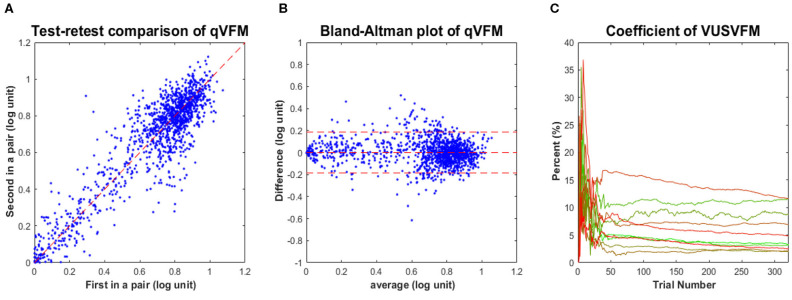
Experimental results III. **(A)** Test-retest comparison of estimated sensitivities from repeated qVFM runs. **(B)** Bland-Altman plot for repeated qVFM runs. **(C)** Coefficient of variability of estimated VUSVFMs (4 runs each) as functions of trial number for the 10 tested eyes.

Test–retest reliability of the qVFM is assessed through analysis of the 4 qVFM runs completed in four sessions. Figure 7A plots estimated sensitivities of the paired qVFM runs from the four independent sessions (2 random pairs of qVFM × 10 eyes × 64 locations = 1,280 data points). The average test–retest correlation for the all possible pairs of VFM estimates was 0.971 (SD = 0.001).

Although test–retest correlation is widely reported as a measure of test–retest reliability, it might not be the most useful way to characterize the reliability of a method (Bland and Altman, 1986). Figure 7B presents a Bland–Altman plot of the difference of the qVFM estimates between all possible pairs of repeated measures against their respective means. The mean and standard deviation of the test–retest difference were 1.3 × 10^−4^ and 0.093 *log(sensitivity)*, respectively. These results suggest that (1) the estimated VFM did not change much over the course of testing sessions, and (2) the test–retest differences between sessions were comparable to the estimated RMSE^eyes^ [0.093 *log(sensitivity)* vs. 0.11 *log(sensitivity)*]. Repeated runs of the qVFM procedure generated quite consistent results, demonstrating its robustness.

To illustrate the convergence of the estimated VUSVFM obtained with the qVFM method, Figure 7C presents the coefficient of variation of VUSVFM estimates as a function of trial number for each eye. The coefficient of variation, also known as relative standard deviation, is defined as the ratio of the standard deviation to the mean:

(9) $$\begin{array}{l} cv_{i}=\frac{\sigma_{i}}{\mu_{i}} \end{array}$$

where σ_*i*_ is the standard deviation of estimated VUSVFM after the *i*-th trial across four runs, and μ_*i*_ is the mean of the estimated VUSVFMs after the *i*-th trial across four runs. A consistent pattern, exhibited in each tested eye, is a decrease of variability with trial number: from close to 35% after 20 trials, to less than 12% after 320 trials.

## Discussion

Visual filed mapping has undergone revolutionary changes over the past 2000 years, particularly with regard to instrumentation, standardization, quantitative assessment, statistical evaluation, optimization of accuracy, precision and efficiency of testing, and distribution of results (Lascaratos and Marketos, 1988; Walsh, 2010). However, the primary method for performing perimetry tests has remained relatively the same for more than 200 years. Thus, it is both a challenge and an opportunity for us to augment current methods by developing new procedures with novel algorithms that would allow more comprehensive and precise identification of damage to the visual field (Thompson and Wall, 2010; Johnson et al., 2011).

We developed the qVFM method to address this technical challenge in mapping visual functions, based on a hybrid Bayesian adaptive testing framework that combines a global module for preliminary assessment of the VFM's shape and a local module for assessing individual VF locations. We first applied the method to assess light sensitivity VFM in an earlier study. In the current study, we extended the method to assess contrast sensitivity of the visual field, and showed that the method can provide an accurate, precise, efficient assessment. Our simulations showed that the average RMSE^simulation^ and SD of the estimated VFM [in log10 units, represent as *log*(*sensitivity*)] after 1,280 trials were 0.037 and 0.032 by the qVFM, and 0.041 and 0.041 by the qFC, respectively. To achieve 0.1 accuracy and 0.1 precision, on average, it took 106 qVFM trials, and 334 qFC trials. Estimates of within-run variability (68.2% HWCIs) were comparable to cross-run variability (SD). For the subjects in our psychophysical experiment, the average HWCI of the qVFM estimates decreased from 0.33 on the first trial to 0.072 after 160 trials, and to 0.060 after 320 trials. The RMSE^eyes^ of the estimates from the qVFM and qFC methods started at 0.26 on the first trial and decreased to 0.12 after 160 qVFM trials and to 0.11 after 320 trials.

In addition to light sensitivity and contrast sensitivity, the qVFM method can be extended to map many other visual functions, such as visual acuity, binocular vision, color vision, temporal frequency, motion sensitivity, reading speed, and crowding maps, with potential clinical signals for monitoring vision loss, evaluating therapeutic interventions, and developing effective rehabilitation for low vision.

The development of the qVFM and other related methods, such as the qCSF, qVA, and qReading methods (Lesmes et al., 2010; Hou et al., 2018; Lesmes and Dorr, 2019; Shepard et al., 2019; Zhao et al., 2019a), makes it possible for us to identify core deficits of functional vision in visual impairments. By measuring performance in a battery of everyday visual tasks on a large group of subjects, we can model their performance in everyday visual tasks with the candidate metrics provided by the tests (e.g., light sensitivity, contrast sensitivity, acuity, reading speed) and identify the most important core metrics. Such core metrics would allow us to better understand visual deficits, to focus on a reduced set of measures while achieving a thorough assessment of residual vision, and to setup portfolio of effective examinations and rehabilitation interventions.

### Mapping Sensitivities With m-AFC Tasks

Earlier adaptive methods focused on targeting pre-defined percent correct performance levels on the empirical psychometric function. Following the development of staircase procedures (Von Békésy, 1947; Wetherill, 1963; Wetherill and Levitt, 1965), the QUEST method (Watson and Pelli, 1983) was a landmark application of Bayesian adaptive inference to measure thresholds. The Bayesian adaptive approach has since been applied to measure empirical thresholds in forced-choice tasks (Watson and Pelli, 1983; King-Smith et al., 1994; King-Smith and Rose, 1997; Snoeren and Puts, 1997; Alcala-Quintana and Garcia-Perez, 2007; García-Pérez and Alcalá-Quintana, 2007).

Previous studies (Leek et al., 1992; Leek, 2001; Alcalá-Quintana and García-Pérez, 2004; Hou et al., 2010) have revealed that the shape of the psychometric function could have a profound impact on the efficiency of adaptive procedures that search optimal stimuli in a two-dimensional stimulus space. In a particular experimental setting, the slope of the *d*′ psychometric function is related to the internal noise distribution and transducer of the observer (Dosher and Lu, 1998; Lu and Dosher, 2008, 2013) and is not easy to manipulate. However, for a single *d*′ psychometric function, it is possible to reduce the guessing rate and increase the slope of the percent correct psychometric function by increasing the number of alternatives in an m-AFC task, and therefore increase the efficiency of the adaptive procedure. The benefit of more alternatives in m-AFC tasks was documented in association with the qCSF method (Hou et al., 2015), and has been extended to the qVFM procedure in this study. It can also be extended to other Bayesian adaptive testing procedures such as QUEST, ZEST, Psi, quick TvC, quick Partial Report, qReading and quick Change-Detection, most of which are based on *d*′ psychometric functions (King-Smith et al., 1994; Kontsevich and Tyler, 1999; Kujala and Lukka, 2006; Lesmes et al., 2006; Baek et al., 2016; Hou et al., 2018; Shepard et al., 2019; Zhang et al., 2019; Zhao et al., 2019b).

### Effects of the Prior

It is well-known that the initial prior probability distribution could change the starting point of parameter estimation and the efficiency of the estimation process (Baek et al., 2016; Gu et al., 2016). For the three simulated observers in the current study, the prior distributions were moderately informative. To illustrate the effects of the prior, we conducted an additional set of simulations with four different prior settings (Table 2): (a) a weakly informative proper prior, (b) a weakly informative but improper prior, (c) a strong informative proper prior, and (d) a strong informative but improper prior.

**Table 2:** The parameters of four prior settings.

**Priors**	**θ_i_**	**EPA**	**EPB**	**EPZ**	**SLA**	**SLB**
WP	θ_i,guess_	72	54	0.6	0.002	0.003
	θ_confidence_	1.7	1.3	1.2	7.0	6.4
WI	θ_i,guess_	66	48	0.9	0.003	0.001
	θ_confidence_	1.7	1.3	1.2	7.0	6.4
SP	θ_i,guess_	72	54	0.6	0.002	0.003
	θ_confidence_	8.4	7.8	6.8	28	25
SI	θ_i,guess_	66	48	0.9	0.003	0.001
	θ_confidence_	8.4	7.8	6.8	28	25

*Unit of EPA, EPB is degree/$\sqrt{log(sensitivity)}$, unit of EPZ is log(sensitivity), unit of SLA, SLB is log(sensitivity)/degree*.

The parameters of the simulated observer were: EPA = 72 (degree/$\sqrt{log\left(sensitivity\right)}$), EPB = 54 (degree/$\sqrt{log\left(sensitivity\right)}$), EPZ = 0.6 (*log*(*sensitivity*)), SLA = 0.002 (*log*(*sensitivity*)/degree), SLB = 0.003 (*log*(*sensitivity*)/degree). The parameter space and the stimulus space remained the same.

Here, we introduce the absolute bias as the index of accuracy. The average absolute bias of the estimated threshold across all locations after the i-th trial can be calculated as:

(10) $$\begin{array}{l} abs\;Bias_{i}=\frac{\sum_{k}|\sum_{j}\left(\tau_{ijk}-\tau_{k}^{true}\right)|}{J\times K} \end{array}$$

where τ_*ijk*_ is the estimated contrast sensitivity in the *k*-th retina location after *i* trials in the *j*-th run, and $\tau_{k}^{true}$ is the true sensitivity of that location.

**Figure 8:**
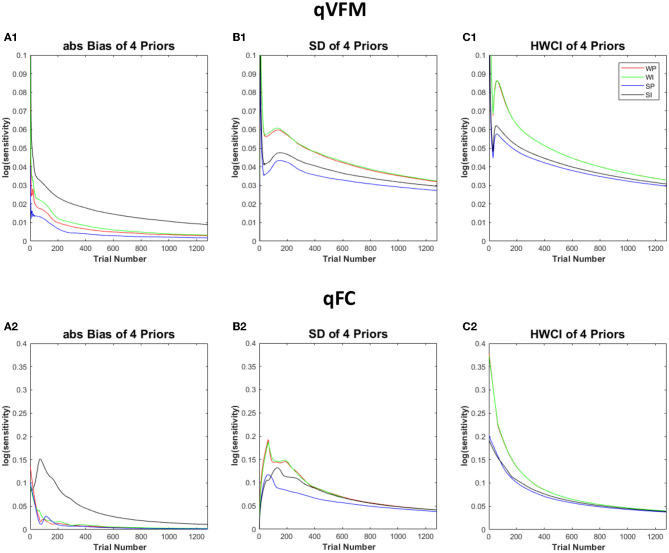
Simulation results III. The performance of the qVFM and qFC procedures in four prior settings. WP, weakly informative proper prior; WI, weakly informative but improper prior; SP, strong informative proper prior; SI, strong informative but improper prior. **(A1, B1, C1)** The average absolute Bias, SD, the average 68.2% HWCI for qVFM procedures in each of four prior settings. **(A2, B2, C2)** The average absolute Bias, SD, the average 68.2% HWCI for qFC procedures in each of four prior settings.

Figure 8 shows the performance of the qVFM and qFC procedures with the four different prior settings. In both the qVFM and qFC procedures, the strong informative proper prior led to the best performance in terms of the average absolute bias, SD and the average 68.2% HWCI. The strong informative improper prior led to the worst average absolute bias and slightly better precision than the weakly informative priors. The weakly informative proper and improper priors exhibited similar performance in all measures, with accuracy between those of the strong informative proper and improper priors, and worse precision comparing to them.

In all four prior settings, the qVFM procedure led to better performance than the qFC procedure. Especially with the strong informative priors, the improper prior made the accuracy of the qFC estimates much worse. The difference of the average absolute bias between the strong informative proper and improper priors was 0.05 for qFC and 0.016 for qVFM after 320 trials. The results suggest that the qVFM method was more robust than the qFC method when the prior was improper.

These results suggest that proper informative prior can speed up the estimation process of the qVFM procedure. We can inform the prior with previous knowledge or pilot data, such as the representative parameters from a particular patient population, or priors derived with the hierarchical adaptive method (Kim et al., 2014; Gu et al., 2016).

## Conclusion

In this study, we implemented the qVFM method to measure contrast sensitivity VFM with a 10-alternative forced-choice paradigm. Detailed assessment of contrast sensitivity across the visual field and other core metrics of functional visual is critical for quantifying the effectiveness of new drugs and rehabilitation therapies. We have tested our method on 10 eyes of five normal observers. Applications of our method to clinical populations may require additional development. Further integrating with other measurements, such as fundus or OCT images, may further improve the efficiency of the qVFM method. The broad adoption of the qVFM method can potentially improve both clinical research and clinical care.

## Data Availability Statement

The datasets generated for this study are available on request to the corresponding author.

## Ethics Statement

The studies involving human participants were reviewed and approved by the Institutional Review Board of the Ohio State University. The patients/participants provided their written informed consent to participate in this study.

## Author Contributions

Z-LL, PX, LL, and DY designed the qVFM algorithms. PX performed simulations, carried out the experiment, and analyzed the data. PX and Z-LL wrote the manuscript with input from all authors. Z-LL and DY supervised the project. All authors contributed to the article and approved the submitted version.

## Conflict of Interest

Z-LL, PX, LL, and DY own intellectual property rights on the qVFM technology and have a pending patent on it. LL and Z-LL have equity interest in Adaptive Sensory Technology, Inc. LL holds employment at AST.

## Supplementary Material

Supplementary Movie 1 A simulated 320-trial sequence of the qVFM and qFC procedures based on the parameters of simulated observer 1. The first row shows the contrast sensitivity VFM of the simulated observer, and the estimated contrast sensitivity VFM's obtained with the qVFM and qFC methods. The second and third rows show the estimated VUSVFM and average HWCI of the estimated contrast sensitivities.
