# Supplementary material for: Mapping the Contrast Sensitivity of the Visual Field With Bayesian Adaptive qVFM
Source: Front Neurosci. 2020 Jul 7;14:665. doi: 10.3389/fnins.2020.00665 (PMC7358309; doi:10.3389/fnins.2020.00665)
Supplement: Supplementary file 2 [file Data_Sheet_1.docx]

# Appendix A: The simulation results of simulated observers 2-3

The estimated contrast sensitivity VFMs of the OS eyes from simulated observers 2-3 are shown in Figure A1 and A2.

**Figure A1**: Simulation results of observer 2: The VFM of the simulated observer (I,1; II,1); The estimated VFM obtained with the qVFM method after 1280 trials (I,2; II,2) and 320 trials (I,3; II,3); The estimated VFM obtained with the qFC method (I,4; II,4); And the corresponding RMSE**^simulation^** (III), standard deviation (IV), and HWCI (V) of the estimates.

**Figure A2**. Simulation results of observer 3. Details are the same as Figure A1.

# Appendix B: The experimental results of subject 2-5

The estimated contrast sensitivity VFMs of the OS and OD eyes from Subject 2-5 are shown in Figure B1 to B4.

**Figure B1.** Experimental results of subject 2 (both OS and OD). The estimated VFMs are presented in the first row with colormaps and second row with numerical values (unit: 100 x $log(sensitivity)$). For each visual field location of the estimated VFM, the 68.2% HWCI is presented in the third row, the standard deviation of the estimated contrast sensitivity from the 4 repeated qVFM assessments and the RMSE**^eyes^** between the qVFM and qFC estimates are presented in the fourth row. The results obtained from OS are displayed in the first and second columns, and OD in the third and fourth columns, respectively. The results from the qVFM and qFC methods are displayed in different columns.

**Figure B2**. Experimental results of subject 3. Details are the same as Figure B1.

**Figure B3**. Experimental results of subject 4. Details are the same as Figure B1.

**Figure B4**. Experimental results of subject 5. Details are the same as Figure B1.

# Appendix C: The qVFM method

The qVFM method consists of three major modules. In the global module, the shape of the VFM is modeled as a tilted elliptic paraboloid function (TEPF) with five parameters (Eq. 1). The score of the VFM at each visual field location represents a measure of visual function in that location. In mapping contrast sensitivity, the score in each location of the VFM represents contrast sensitivity (1/contrast threshold) at a fixed d’ level in that location. Together with a slope of the psychometric function (assumed to be fixed), the VFM can be used to predict the probability of correct optotype identification in every single visual field location. Using Bayesian update and optimal stimulus selection (Kontsevich and Tyler, 1999; Lesmes et al., 2015) , the global module updates the joint posterior distribution of the five parameters, that is, the shape of the VFM, based on subject’s response in each trial.

The switch module evaluates the rate of information gain in the global module and determines when to switch to the local module. At the point of the switch, the module generates a prior distribution of measures of visual function in each visual field location based on the posterior from the global module. In this study, the measure of visual function is the contrast sensitivity at each visual field location.

Using the prior generated by the switch module, the local module provides assessment of visual function in each visual field location using another Bayesian adaptive procedure that determines the order and stimulus of test based on the relative information gain across locations. In mapping contrast sensitivity, we used qFC (Lesmes et al., 2015) as the adaptive procedure to assess visual function at each visual field location and the expected information gain across all visual field locations and stimulus contrast levels to determine the optimal stimulus in each trial.

*The Global Module*

In the global module, we first define a five-dimensional prior probability distribution, in which the five dimensions correspond to the five parameters of the tilted elliptic paraboloid function (TEPF) in mapping visual field (Eq.1). The prior distribution, together with the slope of the psychometric function, completely specifies the probability of response probabilities in the identification task across all the contrast levels and visual field locations of all possible observers. We then define a two-dimensional stimulus space, representing both the spatial locations and contrasts of the stimuli. The optimal stimulus (location and contrast) for the first test is determined based on the prior distribution. Bayes rule is used to update the posterior distribution of the five parameters based on the observer’s response. A new trial starts using the posterior from the previous trial as the prior. The procedure repeats until the switch module decides to switch to the local module.

Setting the prior and stimulus space

Before running the qVFM procedure, a probability density function, $p(\vec{\theta})$, where $\vec{\theta}=\left( EPZ, EPA, EPB, SLA, SLB, \lambda\right),$is defined over the parameter space of the TEPF and decision criterion. Before any data collection (trial *t* = 0), the initial prior distribution *p_t_* _=0_($\vec{\theta}$) represents foreknowledge of model parameters. In addition, a stimulus space, that includes all possible stimulus locations (x, y) and stimulus intensities is defined. With the prior and the stimulus space, we can compute the probability of detecting any stimulus in the visual field for all possible observers based on Eq. 4.

Stimulus selection

In the qVFM, information is quantified by entropy, a measure of uncertainty associated with random variables. The method uses a one-step-ahead search strategy to determine the optimal stimulus in the next trial that would lead to the minimum expected entropy. It first computes (1) the observer’s response probability *P_t+1_(r|s)* in every possible stimulus condition in the next trial based on the current prior, (2) the expected posterior probability distributions for all possible stimuli, and (3) the expected entropy for each possible stimulus.

The entropy of the posterior is defined as:

$H_{t+1}\left( s,r \right)=-\sum_{\vec{\theta}} P_{t+1}\left( \vec{\theta}|s,r \right)\log\left( P_{t+1}(\vec{\theta}|s,r) \right) ,$ (C1)

where *r* represents observer’s response (correct or incorrect) to a test with signal intensity *s*. The expected entropy after a trial with the signal intensity of stimulus, *s,* is calculated as a weighted sum of posterior entropy:

${E[H}_{t+1}\left( s,r \right)]=\sum_{r} H_{t+1}\left( s,r \right)P_{t+1}\left( r | s \right).$ (C2)

The stimulus with the minimum expected entropy is chosen for the next trial:

$s_{t+1}=\arg\min_{s} E[H_{t+1}\left( s \right)]$ . (C3)

This is equivalent to maximizing the expected information gain, quantified as the entropy change between the prior and posterior (Kujala and Lukka, 2006; Lesmes et al., 2006).

Bayesian update

The prior distribution *p_t_(*$\vec{\theta}$*)* in the *t-*th trial is updated to the posterior distribution *p_t_(*$\vec{\theta}$*|s,r_t_)* with the observer’s response *r_t_* (correct or incorrect) to a test with a stimulus, *s,* by Bayes rule:

$P_{t}\left( \vec{\theta} | s,r_{t} \right)=\frac{P\left( r_{t} | \vec{\theta},s \right)P_{t}(\vec{\theta})}{P_{t}\left( r_{t} | s \right)}$ , (C4)

where *θ* represents the parameters of the VFM model, *p_t_(*$\vec{\theta}$*)* is the prior probability function of $\vec{\theta}$. The probability of a response *r_t_* in a given stimulus condition *s*, *p_t_(r_t_|s)*, is estimated by weighting the empirical response probability by the prior:

$P_{t}\left( r_{t} | s \right)=\sum_{\vec{\theta}} [{P\left( r_{t} | \vec{\theta},s \right)P}_{t}(\vec{\theta})]$, (C5)

where *p(r_t_|s,*$\vec{\theta}$*)* is the likelihood of observing response *r_t_* given $\vec{\theta}$ and stimulus s; The posterior *p_t_(*$\vec{\theta}$*|s,r_t_)* following the *t-*th trial serves as the prior *p_t+1_(*$\vec{\theta}$*)* in the next trial:

$P_{t+1}(\vec{\theta})=P_{t}(\vec{\theta}|s,r_{t})$. (C6)

The means of the marginal posterior distributions are used to estimate the parameters of the qVFM model after each trial.

*The Switch Module*

Since TEPF does not provide detailed local measures of the VFM, we designed a switch module to transfer from the global module to the local module for further assessment.

In the global module, the expected information gain, computed as the difference between the entropy of the prior and the expected posterior distribution for each potential stimulus is computed before each trial. The stimulus that would lead to the maximum amount of information gain is used in the next trial. Before each trial, the expected information gain is also used by the switch module to determine the switch point to the local module. Instead of using the maximum expected information gain, the switch module computes the total expected information gain (TEI) from the top 10% potential stimuli. In the beginning, the TEI is high in the global module. With increasing number of trials, the TEI is expected to gradually decrease as the method learns more about the parameters. As the learning saturates over trials, the trend of TEI begins to flatten and may even reverse – that is, the TEI in trial t+1 may be higher than that of its previous trials. In the current implementation, the switch module compares the TEI in trial t+1 with the average TEI of three previous trials, t-2, t-1, and t to determine whether to switch to the local module. The switch happens when the TEI in trial t+1 is higher than the average in trial t-2, t-1 and t.

Upon the switch, the switch module generates a prior distribution of visual function in each visual field location based on the posterior of the parameters in the global module. Specifically, the posterior distribution is sampled repeatedly to generate the prior distribution for each visual field location.

*The Local Module*

The setup of the local module is very similar to that of the global module except the following:

1. Independent parameters in each visual field location: Instead of using five parameters to model visual function across all visual field locations, independent parameters are used to model visual function in each visual field location. In mapping contrast sensitivity, $\tau\left( x,y \right)$is no longer described by Eq. 1, but is rather independent in each location.
2. Independent priors and posteriors: Each visual field location has its independent parameters and therefore independent priors and posteriors. The initial priors in the local module are generated by the switch module.
3. Computing the information gain: The information gain of each location is computed independently, while in the global module the information gains are computed simultaneously across all visual field locations based on the tilted elliptic paraboloid function and the psychometric function. Regardless of the dependency of the computations of information gains, optimal stimulus selection is always based on the total expected entropy across all the visual field locations in both local and global modules. In other words, to select the next test location and stimulus intensity, the expected entropy from all visual field locations are considered.

*Stopping rules*

In the current implementation, the qVFM procedure terminates after a fixed number of trials. Alternatively, the qVFM procedure can stop after it achieves a certain defined objective (e.g., after reaching a criterion level of precision for either the parameters in qVFM or the contrast sensitivity across all visual field locations).
